# The umami receptor T1R1–T1R3 heterodimer is rarely formed in chickens

**DOI:** 10.1038/s41598-021-91728-9

**Published:** 2021-06-10

**Authors:** Yuta Yoshida, Fuminori Kawabata, Shotaro Nishimura, Shoji Tabata

**Affiliations:** 1 Laboratory of Functional Anatomy, Faculty of Agriculture, Kyushu University, Fukuoka, Japan; 2 Department of Food and Life Sciences, Ibaraki University, Ami, Japan; 3 Physiology of Domestic Animals, Faculty of Agriculture and Life Science, Hirosaki University, 3 Bunkyo-cho, Hirosaki, Aomori 036-8561 Japan

**Keywords:** Taste receptors, Animal physiology

## Abstract

The characterization of molecular mechanisms underlying the taste-sensing system of chickens will add to our understanding of their feeding behaviors in poultry farming. In the mammalian taste system, the heterodimer of taste receptor type 1 members 1/3 (T1R1/T1R3) functions as an umami (amino acid) taste receptor. Here, we analyzed the expression patterns of T1R1 and T1R3 in the taste cells of chickens, labeled by the molecular markers for chicken taste buds (vimentin and α-gustducin). We observed that α-gustducin was expressed in some of the chicken T1R3-positive taste bud cells but rarely expressed in the T1R1-positive and T2R7-positive taste bud cells. These results raise the possibility that there is another second messenger signaling system in chicken taste sensory cells. We also observed that T1R3 and α-gustducin were expressed mostly in the vimentin-positive taste bud cells, whereas T1R1 and bitter taste receptor (i.e., taste receptor type 2 member 7, T2R7) were expressed largely in the vimentin-negative taste bud cells in chickens. In addition, we observed that T1R1 and T1R3 were co-expressed in about 5% of chickens' taste bud cells, which express T1R1 or T1R3. These results suggest that the heterodimer of T1R1 and T1R3 is rarely formed in chickens’ taste bud cells, and they provide comparative insights into the expressional regulation of taste receptors in the taste bud cells of vertebrates.

## Introduction

The sense of taste plays an important role in an animal or human’s detection of nutrition and toxic substances in foods, and it is related to feeding behaviors of animals. A greater understanding of the precise molecular mechanisms that underlie the taste-sensing systems in chickens will help improve the feeding strategies used in poultry farming. Umami taste is one of the five basic taste qualities, along with sweet, bitter, sour, and salty, and it is elicited by some l-amino acids and 5′-ribonucleotides in foods. In this study, we focused on the molecular basis of umami taste-sensing systems in chickens.

We reported that chickens prefer food with umami substances over regular feed in a long-term two-feed choice test^1^, but we pointed out that the results on this long-term test may be affected in part by post-ingestive effects. In behavioral tests, chickens showed little or no preference for umami taste solutions^2–4^. The low behavioral preference for umami taste solutions in chickens is unlike the preferences that have been reported in mice, rats and cows, which show a significant preference for umami taste solutions^5–7^. In a conditioned taste aversion (CTA) test, chickens learned to avoid umami taste solution^4^, and we thus speculated that chickens have an orosensory perception system for umami taste.

In the mammalian taste-sensing system, the heterodimer of taste receptor type 1 members 1 and 3 (T1R1 and T1R3) functions as the umami taste receptor^8^. These subunits are required for the formation of umami taste receptors in the heterologous expression systems^8^, and mice that lack either T1R1 or T1R3 genetically show dramatically reduced preference for and sensitivity to umami taste^5,9,10^. *T1R1* and *T1R3* are largely co-expressed with each other in the taste cells of mice^11^, and single-cell reverse transcription-polymerase chain reaction (RT-PCR) revealed that 57% of *T1R1*-expressing taste cells of mice expressed *T1R3*^10^.

In the chicken genome, both *T1R* genes for the umami taste receptor (T1R1 and T1R3) have been identified, but *T1R2* gene for the sweet taste receptor is lacking^12^. The mRNA of both *T1R1* and *T1R3* is expressed in the taste tissues of chickens^1,4^, and a heterologous expression analysis demonstrated that chicken T1R1/T1R3 is functional and responds to l-amino acids, i.e., l-alanine and l-serine^13^. Molecular markers for chicken taste buds have been identified: vimentin and α-gustducin, which label a subset of taste bud cells in all chicken taste buds^14–16^. We showed that T1R1 and bitter taste receptor, taste receptor type 2 member 7 (T2R7) are specifically expressed in the taste bud cells of chickens^17^.

In the present study, we newly generated anti-chicken T1R3 antiserum, and we analyzed the expression patterns of the umami taste receptor subunits T1R1 and T1R3 in the taste bud cells of chickens. The results revealed that T1R1 is expressed mainly in the α-gustducin-negative taste bud cells whereas T1R3 is expressed in some α-gustducin-positive taste bud cells; T1R1 is expressed mainly in the chicken vimentin-negative taste bud cells and T1R3 is expressed largely in the vimentin-positive taste bud cells. In addition, we revealed differing expression patterns of T1R1 and T1R3 in the chicken taste bud cells by a double fluorescence immunohistochemistry (IHC). It is possible that this expression pattern is consistent with chickens' low preference for umami taste solutions, and our knowledge of the pattern in chickens contributes to the understanding of the evolution of taste-sensing systems in vertebrates and the regulation of taste receptor expression.

## Results

### Immunohistochemistry revealed the expression of T1R3 in the taste bud cells of chickens

**Figure 1:**
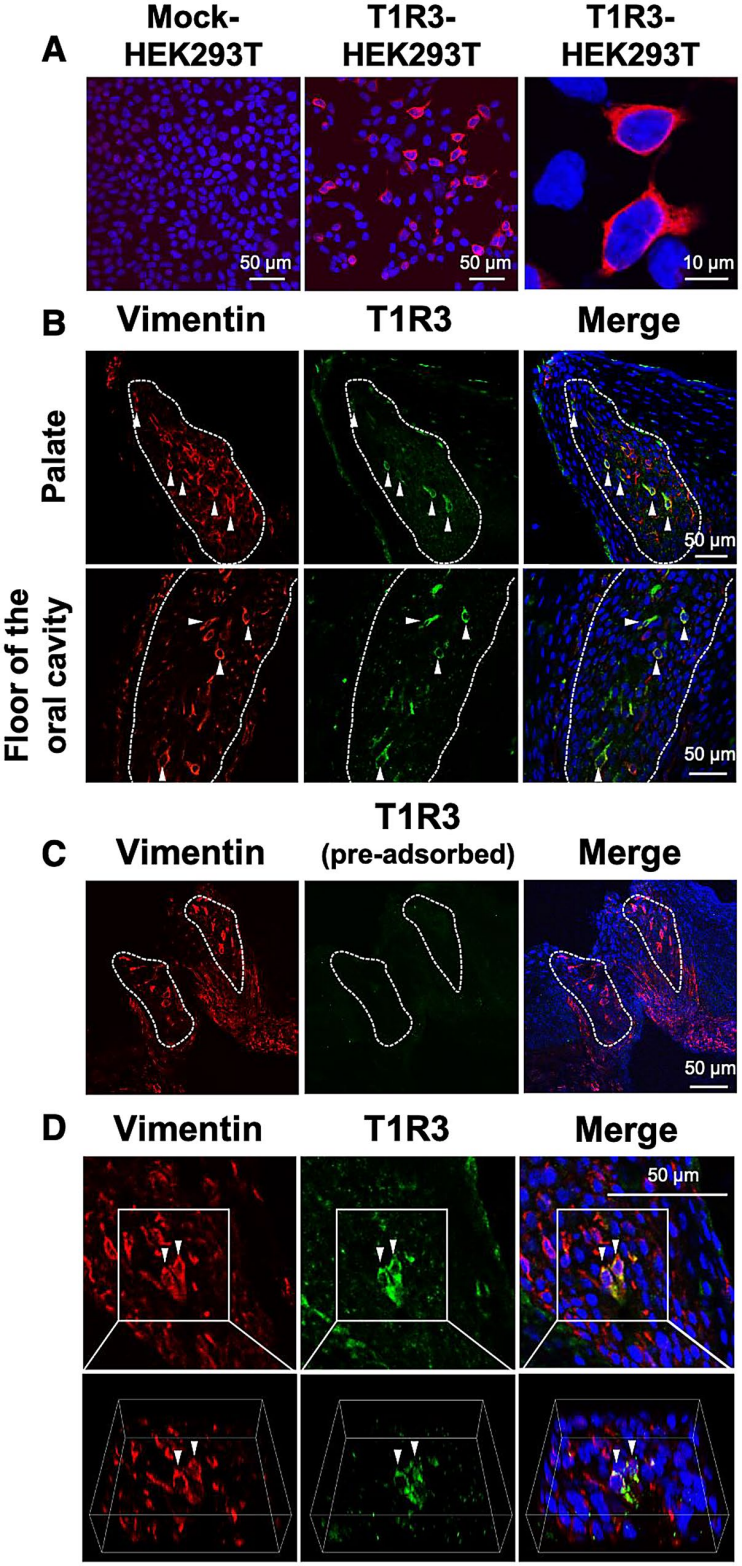
(**A**) HEK293T cells transiently transfected with empty vector pcDNA5/FRT (mock) were not labeled by the chicken T1R3 antiserum generated herein, but HEK293T cells transiently transfected with chicken *T1R3*/pcDNA5/FRT were labeled by the antiserum (*red)*. Nuclei were stained with DAPI (*blue*). (**B**) Distribution of vimentin (*red*) and T1R3 (*green*) immunosignals in the oral tissues of 10-week-old chickens in the palate and floor of the oral cavity. *White dots* outline the taste buds labeled by the molecular marker for the taste buds of chickens, vimentin. *Arrowheads* indicate taste bud cells labeled by both vimentin and T1R3. Nuclei were stained with DAPI (*blue*). The images were representative and 35 images were analyzed for co-expression of T1R3 and vimentin. (**C**) Diminished T1R3 (*green*) immunosignal by pre-incubation with the excess synthetic peptide in the taste bud cells, labeled by vimentin (*red*). Nuclei were stained with DAPI (*blue*). (**D**) Distribution of vimentin (*red*) and T1R3 (*green*) immunosignals in the oral tissues in the floor of the oral cavity. The 3-D images (*below*) could construct the structure of the taste sensory cells co-expressing vimentin and T1R3 (*arrowheads*). Nuclei were stained with DAPI (*blue*).

Firstly, the specificity of the chicken T1R3 antiserum newly generated in this study was investigated. We observed that HEK293T cells transfected with the empty vector pcDNA5/FRT were not labeled, whereas the HEK293T cells transfected with chicken *T1R3*/pcDNA5/FRT were specifically labeled (Fig. 1A). Using this antiserum, we confirmed the expression of T1R3 in the chicken taste buds labeled by the molecular marker for chicken taste buds, i.e., vimentin (Fig. 1B). It is notable that T1R3 was expressed in the vimentin-positive taste bud cells (Fig. 1B,D). The specificity of this antiserum was further validated by a pre-adsorption test. We confirmed that the specific immunosignals within chicken taste buds diminished by the incubation of the antiserum with the excess peptide (Fig. 1C).

### T1R1 and T1R3 were separately expressed in the α-gustducin-negative or -positive taste bud cells of chickens

**Figure 2:**
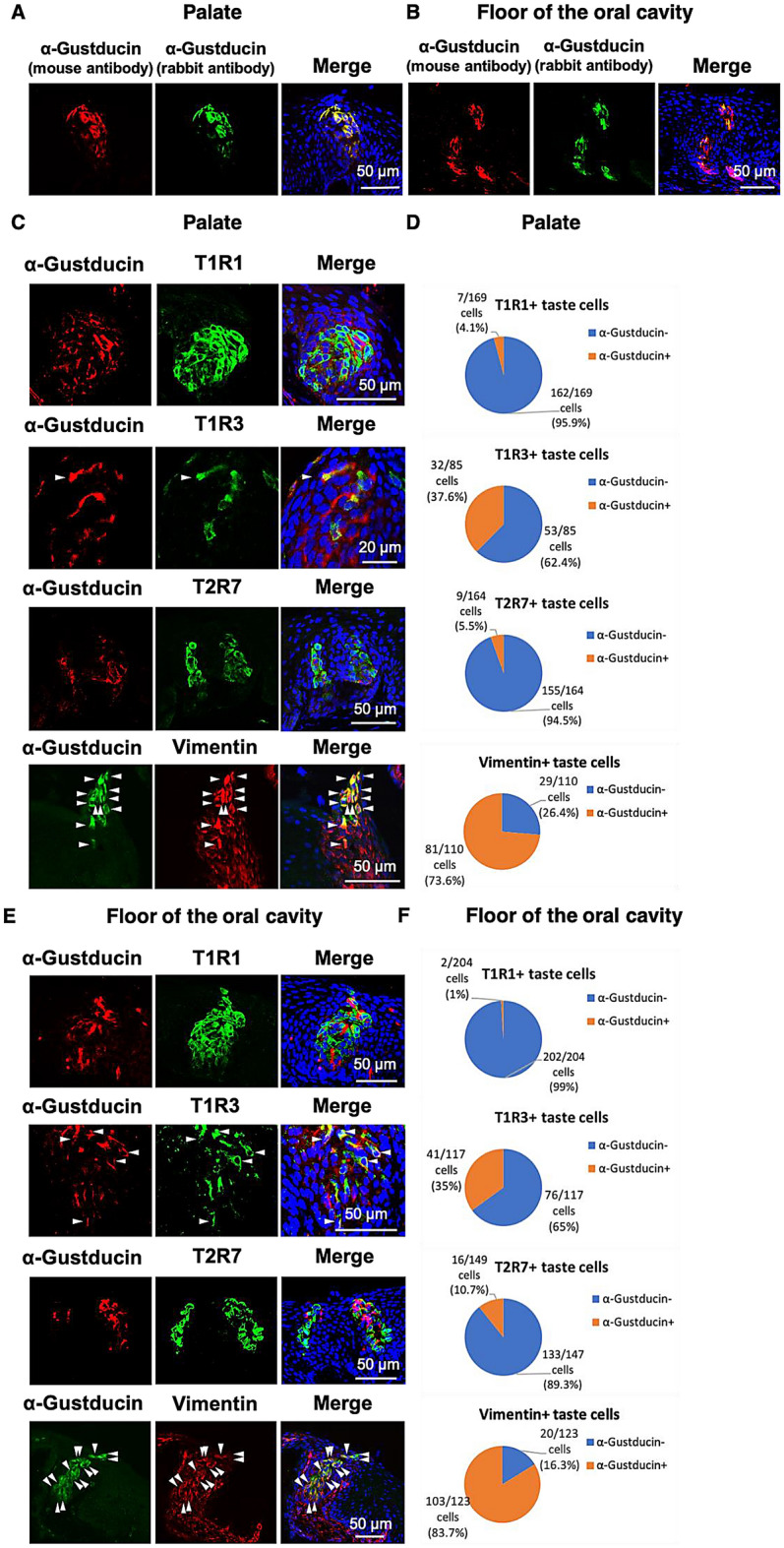
(**A**, **B**): The distributions of α-gustducin, labeled by the antiserum generated herein (host species: mouse) (*red*) and α-gustducin, labeled by the antiserum (host species: rabbit) (*green*) immunosignals in the oral tissues in the palate (A) or floor of the oral cavity (B) of 7-day-old chickens. Nuclei were stained with DAPI (*blue*). (**C**, **E**): The distributions of α-gustducin (*red*) and T1R1, T1R3, or T2R7 (*green*) immunosignals or α-gustducin (*green*) and vimentin (*red*) immunosignals in the oral tissues in the palate (C) or floor of the oral cavity (E) of 7-day-old chickens. *Arrowheads:* Taste bud cells labeled by both α-gustducin and T1R3 or both α-gustducin and vimentin. Nuclei were stained with DAPI (*blue*). The images were representative and 9 (T1R1), 20 (T1R3), 10 (T2R7), and 16 (vimentin) images were analyzed respectively. (**D**, **F**) Ratio of α-gustducin^+^ cells and α-gustducin^−^ cells in the T1R1^+^, T1R3^+^, T2R7^+^, or vimentin^+^ taste bud cells in the palate (**D**) and floor of the oral cavity (**F**).

We generated a new antiserum for chicken α-gustducin (host species: mouse) to analyze co-expression of T1R1, T1R3, and T2R7 with α-gustducin in the taste buds of chickens. We already had the antiserum for chicken α-gustducin (host species: rabbit). Therefore, to check the specificity of the new antiserum, we confirmed that these antisera, generated from rabbit or mouse, specifically labeled same taste bud cells of chickens (Fig. 2A,B). We also tested the specificity of the antiserum by conducting a pre-adsorption test (data not shown).

Using the new antiserum, we observed that T1R1 was expressed mainly in the α-gustducin-negative taste bud cells (palate: 95.9%, floor of the oral cavity: 99%), whereas the T1R3 was also partly expressed in the α-gustducin-positive taste bud cells (palate: 37.6%, floor of the oral cavity: 35%) (Fig. 2C–F). T2R7 was expressed mainly in the α-gustducin-negative taste bud cells (palate: 94.5%, floor of the oral cavity: 89.3%), indicating that T1R1 and T2R7 are expressed mainly in the vimentin-negative and α-gustducin-negative taste bud cells of chickens^17^. Vimentin was expressed largely in the α-gustducin-positive taste bud cells (palate: 73.6%, floor of the oral cavity: 83.7%), as reported in the previous study^16^.

### T1R1 and T1R3 were separately expressed in the vimentin-negative or -positive taste bud cells

**Figure 3:**
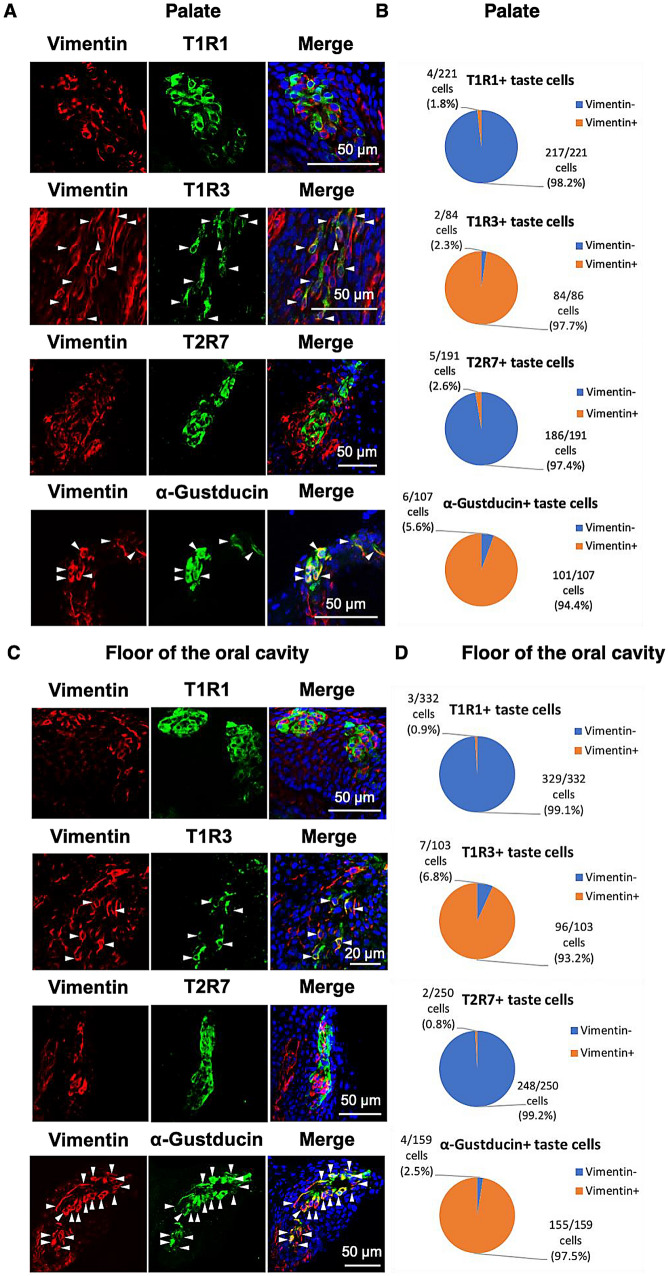
(**A**, **C**) The distributions of vimentin (*red*) and T1R1, T1R3, T2R7, or α-gustducin (*green*) immunosignals in the oral tissues in the palate (**A**) or floor of the oral cavity (**C**) of 7-day-old chickens. *Arrowheads:* Taste bud cells labeled by both vimentin and T1R3 or vimentin and α-gustducin. Nuclei were stained with DAPI (*blue*). The images were representative and 22 (T1R1), 32 (T1R3), 19 (T2R7), and 16 (α-gustducin) images were analyzed respectively. (**B**, **D**) The ratio of vimentin^+^ cells and vimentin^−^ cells in the T1R1^+^, T1R3^+^, T2R7^+^, or α-gustducin^+^ taste bud cells in the palate (**B**) and floor of the oral cavity (**D**) are shown.

We observed that most of the T1R1-expressing taste bud cells were vimentin-negative (palate: 98.2%, floor of the oral cavity: 99.1%), whereas the T1R3-expressing taste bud cells were mostly vimentin-positive (palate: 97.7%, floor of the oral cavity: 93.2%) (Fig. 3). These results suggested that in chickens, T1R1 is expressed mainly in the vimentin-negative taste bud cells and T1R3 is expressed largely in the vimentin-positive taste bud cells. Further analyses also demonstrated that in chickens, the bitter taste receptor T2R7 is expressed mainly in the vimentin-negative taste bud cells (palate: 97.4%, floor of the oral cavity: 99.2%), and α-gustducin is expressed mostly in the vimentin-positive taste bud cells (palate: 94.4%, floor of the oral cavity: 97.5%) (Fig. 3). A summary of the possible co-expression patterns of vimentin, α-gustducin, T1R1, T1R3, and T2R7 in the taste buds of chickens is presented in Supplementary Fig. S3).

### T1R1 and T1R3 were separately expressed in the taste bud cells of chickens

**Figure 4:**
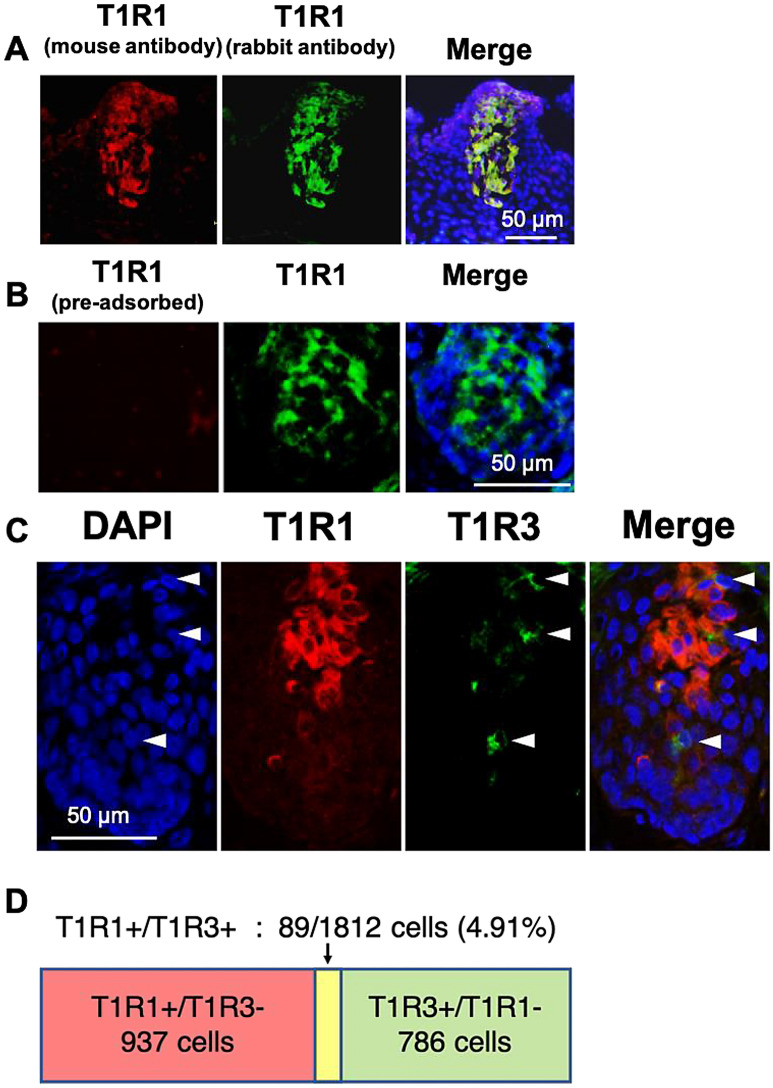
(**A**) The distributions of T1R1, labeled by the antiserum generated herein (host species: mouse) (*red*) and T1R1, labeled by the antiserum (host species: rabbit) (*green*) immunosignals in the oral tissues in the floor of the oral cavity of 6-day-old chicks. Nuclei were stained with DAPI (*blue*). (**B**) Diminished T1R1 (*red*) immunosignal by pre-incubation with the excess synthetic peptide in the taste bud cells, labeled by another T1R1 antiserum (*green*). Nuclei were stained with DAPI (*blue*). (**C**) The distributions of T1R1 (*red*) and T1R3 (*green*) immunosignals in the oral tissues in the palate of 6-day-old chicks. Nuclei were stained with DAPI (*blue*). *Arrowheads:* Taste bud cells labeled by T1R3. The images were representative and 35 images were analyzed. (**D**) The number and ratio of T1R1^+^/T1R3^+^ cells (double-positive cells) among the T1R1^+^ or T1R3^+^ taste bud cells in the palate and floor of the oral cavity.

To analyze the co-expression pattern of T1R1 and T1R3, we newly generated an antiserum for chicken T1R1 (host species: mouse). We confirmed that the antisera for chicken T1R1, generated from rabbit or mouse, specifically labeled same taste bud cells of chickens (Fig. 4A). We further verified the specificity of the antiserum by conducting a pre-adsorption test (Fig. 4B).

Using the new antiserum, we observed that T1R1 was seldom expressed in the T1R3-positive taste bud cells (palate: 8.4%, floor of the oral cavity: 9.03%), and T1R3 was also rarely expressed in the T1R1-positive taste bud cells (palate: 8.91%, floor of the oral cavity: 12.3%) (Supplementary Fig. S1D). Taken together, we observed that the double-positive taste cells, which co-express T1R1 and T1R3, were only 4.91% among the taste cells, which express T1R1 or T1R3 (Fig. 4C,D).

## Discussion

In mammals, the heterodimer of T1R1 and T1R3 acts as an umami (amino acid) taste sensor^8^. It was reported that chicken T1R1/T1R3 heterodimer is also activated by some l-amino acids such as l-alanine and l-serine^13^. T1R1 and T1R3 alone do not function as an umami taste receptor in the heterologous expression systems^8^, and mice lacking either T1R1 or T1R3 show a markedly reduced preference for umami taste^5,9,10^. In an earlier investigation, we observed that umami taste receptor subunit T1R1 was specifically expressed in the taste bud cells of chickens^17^.

In the present study, we generated a specific antiserum for another umami taste receptor subunit (chicken T1R3) to understand the molecular mechanisms for umami taste detection in the taste bud cells of chickens. Firstly, we found that T1R1 and T2R7 receptors were found in vimentin-negative and α-gustducin-negative taste sensory cells. These results raised the possibility that there is another second messenger signal system in chicken taste sensory cells that has not yet been identified. In fact, most *T1R1*-expressing cells expressed *gustducin* in mice^10^. Then, our analyses revealed that T1R1 is expressed mainly in the vimentin-negative taste bud cells of chickens, whereas T1R3 was expressed largely in the chicken vimentin-positive taste bud cells. In addition, we observed that T1R1 and T1R3 were co-expressed in less than 5% of chickens' taste cells expressing T1R1 or T1R3, whereas in mice, single-cell RT-PCR analyses showed that 12 of 21 (57%) T1R1-expressing cells expressed T1R3^10^. These data showed the possibility that the molecular mechanisms of umami taste detection differ between mammals and birds.

In two-bottle preference tests, chickens showed no behavioral preference for umami taste solutions^2,3^ in contrast to mice, rats, and cows^5–7^. Our previous brief-access tests also demonstrated that chickens showed little or no preference for umami taste solutions^4^. In our earlier study using a two-feed choice test, chickens showed a preference for umami-flavored feed over the regular feed, but we noted that the results of this long-term behavioral paradigm may reflect post-ingestive effects^1^. These behavioral data suggest that chickens have only a faint behavioral preference for umami taste solutions, unlike mice. Our present findings revealed the possibility that the heterodimer of T1R1/T1R3 rarely exists in the taste bud cells of chickens, and this could explain the molecular mechanisms underlying the weak behavioral preference for umami taste solutions in chickens. On the other hand, it is noted that T1R1 and T1R3 are co-expressed in about 5% of chickens' taste cells, which express T1R1 or T1R3, and that the other umami taste receptor candidates, metabotropic glutamate receptor (*mGluR1* and *mGluR4*) are expressed in the oral tissues of chickens^1,4^. Thus, the taste cells, which express T1R1/T1R3 or mGluRs, could be involved in the umami taste detection in chickens, previously shown by the conditioned taste aversion test^4^.

The identities of amino acid sequences of chicken T1R1 and T1R3 compared to humans are only 47% in T1R1 and 43% in T1R3, respectively. Despite the low identities of amino acid sequences, all amino acid residues responsible for l-glutamate recognition (T149, S172, D192, Y220, and E301 in human T1R1) and two of four amino acid residues responsible for IMP recognition (H72 and R277 in human T1R1) are conserved in chicken T1R1^18^. In fact, it has been reported that chicken T1R1/T1R3 act as an amino acid taste receptor^13^. Our previous study has shown that mRNA of *T1R1* and *T1R3* are broadly expressed in the gastrointestinal tract^1^. Therefore, it is possible that the heterodimer of T1R1/T1R3 functions in the extra-oral tissues in chickens. On the other hand, it has been reported that mouse and human T1R3 are activated by high concentration of sucrose and calcium, respectively^9,19^. Chickens show behavioral response to high concentration of sucrose^3,4^, and have calcium appetite^20^. Therefore, T1R3 alone could function as high concentration of sweet and calcium taste receptor in the taste buds in chickens. It has been reported that mGluR1 and mGluR4 are also involved in l-amino acid detection and IMP detection in the taste cells in mice^21^. Thus, it is possible that these mGluRs could function as amino acid and IMP receptors in the oral tissues in chickens.

In conclusion, we observed that the umami taste receptor subunit T1R1 was expressed mainly in the vimentin-negative taste bud cells, and the umami taste receptor subunit T1R3 was expressed mostly in the vimentin-positive taste bud cells in chickens. We also observed that T1R1 and T1R3 were rarely co-expressed in the taste bud cells of chickens, and this could be related to chickens’ weak behavioral preference for umami taste solutions. Our findings also suggest that chickens have molecular mechanisms for umami taste detection that differ from those of mammals.

## Methods

### Animals and tissue collection

Rhode Island Red (RIR) strain chicks were obtained from the National Livestock Breeding Center’s Okazaki station (Okazaki, Japan), and the chicks and their offspring were used for this experiment. Zero- to 2-week-old chicks were used for molecular cloning and the determination of the nucleotide sequence of RIR T1R3. Ten-week-old chicks, 6-day-old chicks, and 7-day-old chicks were used for IHC. Tissue collection and preparation of frozen sections were performed as described previously with some modification^17^. The chicks were sacrificed with an overdose of pentobarbital sodium solution, and the palate and floor of the oral cavity were excised. For IHC analyses, the tissues were embedded in Tissue-Tek O.C.T. Compound (Sakura Finetek Japan, Tokyo), and freshly frozen at − 80 °C. Frozen sections were cut at 8–10-µm thickness on a Leica CM1850 cryostat and CM1900 cryostat (Leica Instruments, Nussloch, Germany) and mounted on Matsunami adhesive silane (MAS)-coated glass slides (Matsunami Glass, Osaka, Japan). This study was carried out according to the Guide for Animal Experiments issued by Kyushu University, the Law Concerning the Human Care and Control of Animals (Law No. 105; October 1, 1973), the Japanese Government Notification on the Feeding and Safekeeping of Animals (Notification No. 6; March 27, 1980), and the ARRIVE guidelines and approved by the committee for Laboratory Animal Care and Use at Kyushu University (approval no. A30-335-1) and Ibaraki University with the guidelines of the Experimental Animal Committee (approval no. 20200).

### Chicken T1R3 construction

Total RNA was isolated from the palate of chicks with the use of ISOGEN II (Nippon Gene, Tokyo), and first-strand cDNA was synthesized by reverse transcription using the PrimeScript RT reagent kit with gDNA Eraser (Takara Bio, Otsu, Japan), following the manufacturer’s protocol. The deduced open reading frame (ORF) of chicken *T1R3* was amplified and sequenced.

The polymerase chain reaction (PCR) primers were designed with the use of the U.S. National Center for Biotechnology Information (NCBI) nucleotide database and are as follows. Primer forward: 5′-gtttaaacttaagctttcccctccaaacagggagatta-3′ and primer reverse: 5′-cagcgggtttaaacgggcccttgagtctgcaccacagctta-3′. The PCR products were subcloned into mammalian expression vector pcDNA5/FRT (Life Technologies, Carlsbad, CA) using the In-Fusion HD Cloning Kit (Takara Bio). The entire sequence of RIR *T1R3* was determined (Supplementary Fig. S2).

### Antibodies

The primary antibodies used were custom-made rabbit polyclonal anti-chicken T1R1 antiserum (residues 18–28, RPSPAEPRDGA)^17^, custom-made mouse polyclonal anti-chicken T1R1 antiserum (residues 18–28, RPSPAEPRDGA) (newly generated in this study, Scrum Inc., Tokyo), custom-made rabbit polyclonal anti-chicken T1R3 antiserum (residues 167–179, SEKLSNKELYPSF) (newly generated in this study, Scrum Inc., Tokyo), custom-made rabbit polyclonal anti-chicken T2R7 antiserum (residues 171–184, GIFWKTNEEIRKHF)^17^, rabbit polyclonal anti-chicken α-gustducin antiserum^14^, custom-made mouse polyclonal anti-chicken α-gustducin antiserum (residues 95–106, YENPARIEDERK) (newly generated in this study, Scrum), and mouse monoclonal anti-vimentin antibody (V9; Thermo Fischer Scientific, Waltham, MA)^15–17^. The secondary antibodies used were Alexa Fluor 488 goat anti-rabbit IgG (1:500), Alexa Fluor 594 goat anti-rabbit IgG (1:500), and Alexa Fluor 594 goat anti-mouse IgG (1:500) (Thermo Fischer Scientific).

### Pre-adsorption test

The custom-made rabbit polyclonal anti-chicken T1R3 antiserum, mouse polyclonal anti-chicken α-gustducin antiserum, and mouse polyclonal anti-chicken T1R1 antiserum, newly generated in this study, were pre-incubated with 0.8 mg/ml of excess peptides (T1R3: SEKLSNKELYPSF, α-gustducin: YENPARIEDERK, T1R1: RPSPAEPRDGA, Scrum) in phosphate-buffered saline (PBS) overnight at room temperature (RT). An IHC examination of frozen sections was performed using these pre-adsorbed antibodies with added 1% normal goat serum (NGS) (Vector Laboratories, Burlingame, CA) and 0.2% Triton X-100.

### IHC of HEK293T cells

Culture and transfection of human embryonic kidney 293 T (HEK293T) cells were conducted as described previously^22^. HEK293T cells were maintained in Dulbecco's modified Eagle’s medium (DMEM high glucose, FujiFilm Wako Pure Chemical Corp., Osaka, Japan) containing 10% fetal bovine serum (FBS, GE Healthcare, Buckinghamshire, UK) and penicillin–streptomycin solution (1:100) (FujiFilm Wako) at 37 °C in 5% CO_2_.

HEK293T cells were transfected with either empty vector pcDNA5/FRT or chicken *T1R3*/pcDNA5/FRT by using ScreenFect A (FujiFilm Wako) on coverslips coated with poly-d-lysine (0.1 mg/mL). After transfection, the cells were incubated for 48 h at 37 °C in 5% CO_2_. The HEK293T cells were then washed with PBS and fixed with − 20 °C methanol for 10 min. After another wash with PBS, non-specific staining was blocked by 3% NGS/PBS for 30 min at RT. The cells were then incubated with the rabbit anti-chicken T1R3 antiserum (1:2000) in 1% NGS/PBS overnight at 4 °C.

The cells were washed with PBS and incubated with Alexa Fluor 594 goat anti-rabbit IgG (1:500) in 1% NGS/PBS for 45 min at RT. After the cells were washed with PBS, they were mounted with VECTASHIELD with DAPI (Vector Laboratories). A confocal laser scanning microscope system (A1R; Nikon Instech, Tokyo) with NIS-Element AR3.2 was used for the observation.

### IHC of frozen sections

A double-fluorescence IHC examination of frozen sections was performed as shown in the previous study^17^. The sections were fixed with acetone for 10 min at 4 °C and dried for 60 min at RT. After rehydration with PBS, non-specific staining was blocked by 3% NGS/PBS with Triton X-100 (PBS-X) for 30 min at RT. The sections were then incubated with the primary antibodies in 1% NGS/PBS-X overnight at 4 °C. The sections were rinsed with PBS and incubated with the secondary antibodies in 1% NGS/PBS-X for 60 min at RT. Following rinses with PBS, the sections were mounted using VECTASHIELD with DAPI. The same confocal laser scanning microscope system (A1R) with NIS-Element AR3.2, a fluorescence microscope (Keyence, BZ-8000, Osaka, Japan), and an AxioCam MRm camera (Zeiss, Gottingen, Germany) with the ZEN 2.3 (blue edition) software (Zeiss) were used for the observations.

### Quantifications of taste bud cells

The taste bud cells in the palate and floor of the oral cavity of chickens were quantified. These data were collected from the frozen taste tissue sections of three to four individual 6-day-old and 7-day-old male chicks. The taste bud cells labeled by T1R1, T1R3, T2R7, vimentin, or α-gustducin were quantified. The quantification was carried out manually using photomicrographs obtained from the confocal laser scanning microscope and fluorescent microscope. To ensure consistency among the groups, the same investigator performed each quantification.

## Supplementary Information

Supplementary Information 1.

Supplementary Information 2.

Supplementary Information 3.

## References

[1] Yoshida Y, Kawabata Y, Kawabata F, Nishimura S, Tabata S (2015). Expression of multiple umami taste receptors in oral and gastrointestinal tissues, and umami taste synergism in chickens. Biochem. Biophys. Res. Commun..

[2] Urata K, Manda M, Watanabe S (1992). Behavioral study on taste responses of hens and female Japanese quails to salty, sour, sweet, bitter and umami solutions. Anim. Sci. Technol. (Jpn.).

[3] Cheled-Shoval SL, Reicher N, Niv MY, Uni Z (2017). Detecting thresholds for bitter, umami, and sweet tastants in broiler chicken using a 2-choice test method. Poult. Sci..

[4] Yoshida Y, Kawabata F, Kawabata Y, Nishimura S, Tabata S (2018). Short-term perception of and conditioned taste aversion to umami taste, and oral expression patterns of umami taste receptors in chickens. Physiol. Behav..

[5] Damak S (2003). Detection of sweet and umami taste in the absence of taste receptor T1r3. Science.

[6] Inui-Yamamoto C (2017). Taste preference changes throughout different life stages in male rats. PLoS ONE.

[7] Manda M, Urata K, Noguchi T, Watanabe S (1996). Behavioral study on taste responses of cattle to salty, sour, sweet, bitter, umami and alcohol solutions. Anim. Sci. Technol. (Jpn.).

[8] Nelson G (2001). Mammalian sweet taste receptors. Cell.

[9] Zhao G (2003). The receptors for mammalian sweet and umami taste. Cell.

[10] Kusuhara Y (2013). Taste responses in mice lacking taste receptor subunit T1R1. J. Physiol..

[11] Kim MR (2003). Regional expression patterns of taste receptors and gustducin in the mouse tongue. Biochem. Biophys. Res. Commun..

[12] Shi P, Zhang J (2006). Contrasting modes of evolution between vertebrate sweet/umami receptor genes and bitter receptor genes. Mol. Biol. Evol..

[13] Baldwin MW (2014). Evolution of sweet taste perception in hummingbirds by transformation of the ancestral umami receptor. Science.

[14] Kudo K, Wakamatsu K, Nishimura S, Tabata S (2010). Gustducin is expressed in the taste buds of the chicken. Anim. Sci. J..

[15] Rajapaksha P (2016). Labeling and analysis of chicken taste buds using molecular markers in oral epithelial sheets. Sci. Rep..

[16] Venkatesan N (2016). Distribution of alpha-gustducin and vimentin in premature and mature taste buds in chickens. Biochem. Biophys. Res. Commun..

[17] Yoshida Y (2019). Bitter taste receptor T2R7 and umami taste receptor subunit T1R1 are expressed highly in vimentin-negative taste bud cells in chickens. Biochem. Biophys. Res. Commun..

[18] Zhang F (2008). Molecular mechanism for the umami taste synergism. Proc. Natl. Acad. Sci..

[19] Tordoff MG, Alarcón LK, Valmeki S, Jiang P (2012). T1R3: a human calcium taste receptor. Sci. Rep..

[20] Taher AI, Gleaves EW, Beck M (1984). Special calcium appetite in laying hens. Poult. Sci..

[21] Choudhuri SP, Delay RJ, Delay ER (2016). Metabotropic glutamate receptors are involved in the detection of IMP and L-amino acids by mouse taste sensory cells. Neuroscience.

[22] Dey B (2017). Identification of functional bitter taste receptors and their antagonist in chickens. Biochem. Biophys. Res. Commun..

