# Supplementary material for: The umami receptor T1R1–T1R3 heterodimer is rarely formed in chickens
Source: Sci Rep. 2021 Jun 10;11:12318. doi: 10.1038/s41598-021-91728-9 (PMC8192514; doi:10.1038/s41598-021-91728-9)
Supplement: Supplementary file 1 — Supplementary Information 1. [file 41598_2021_91728_MOESM1_ESM.pdf]

The umami receptor T1R1-T1R3 heterodimer is rarely formed in chickens

Yuta Yoshida[1,2], Fuminori Kawabata[1,3*], Shotaro Nishimura[1], Shoji Tabata[1]

[1]Laboratory of Functional Anatomy, Faculty of Agriculture, Kyushu University, Fukuoka, Japan

[2]Department of Food and Life Sciences, Ibaraki University, Ami, Japan

[3]Physiology of Domestic Animals, Faculty of Agriculture and Life Science, Hirosaki University, Hirosaki, Japan

***Corresponding author**: Dr. Fuminori Kawabata, Physiology of Domestic Animals, Faculty of Agriculture and Life Science, Hirosaki University, 3 Bunkyo-cho, Hirosaki, Aomori 036-8561, Japan. Tel.: +81-172-39-3805. Fax: +81-172-39-3805.
Email: kawabata@hirosaki-u.ac.jp

A

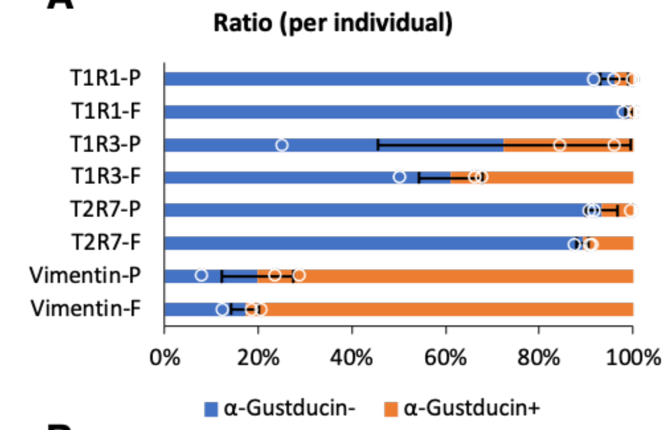

	n number	bud number	cell number
T1R1-P	3	4	169
T1R1-F	3	5	204
T1R3-P	3	10	85
T1R3-F	3	9	117
T2R7-P	3	6	164
T2R7-F	3	4	147
Vimentin-P	3	10	110
Vimentin-F	3	6	123

B

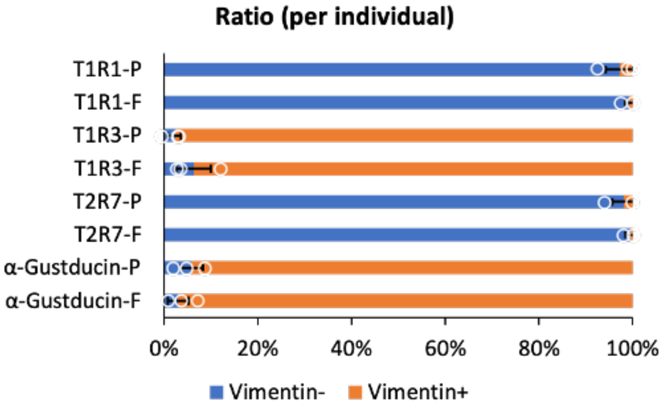

	n number	bud number	cell number
T1R1-P	3	8	221
T1R1-F	3	14	332
T1R3-P	3	12	86
T1R3-F	3	19	103
T2R7-P	3	10	191
T2R7-F	3	9	250
α-Gustducin-P	3	10	107
α-Gustducin-F	3	6	159

C

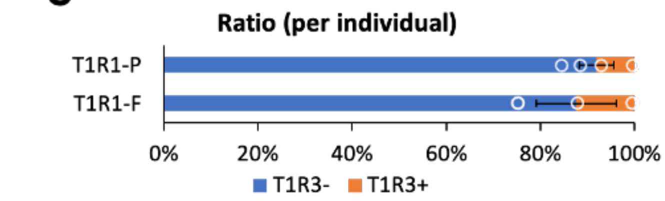

	n number	bud number	cell number
T1R3-P	4	24	583
T1R3-F	3	11	443

D

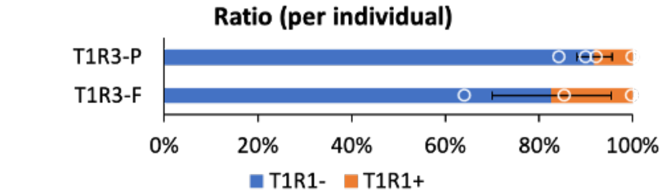

	n number	bud number	cell number
T1R1-P	4	24	550
T1R1-F	3	11	325

Supplementary Figure S1

Supplementary Fig. S1. A: Ratios of α-gustducin^{-} or α-gustducin^{+} cells in the T1R1^{+} cells in the palate (T1R1-P) and floor of oral cavity (T1R1-F), T1R3^{+} cells in the palate (T1R3-P) and floor of oral cavity (T1R3-F), T2R7^{+} cells in the palate (T2R7-P) and floor of oral cavity (T2R7-F), and vimentin^{+} cells in the palate (Vimentin-P) and floor of oral cavity (Vimentin-F). Individual values for each chick were shown as white open circles. Values are the means ± standard errors (SE). Absolute n number, number of taste buds, and number of cells were listed in the table (*right*). **B:** Ratios of vimentin^{-} or vimentin^{+} cells in the T1R1^{+} cells in the palate (T1R1-P) and floor of oral cavity (T1R1-F), T1R3^{+} cells in the palate (T1R3-P) and floor of oral cavity (T1R3-F), T2R7^{+} cells in the palate (T2R7-P) and floor of oral cavity (T2R7-F), and α-gustducin^{+} cells in the palate (α-gustducin-P) and floor of oral cavity (α-gustducin-F). Individual values for each chick were shown as white open circles. Values are the means ± SE. Absolute n number, number of taste buds, and number of cells were listed in the table (*right*). **C:** Ratios of T1R3^{-} or T1R3^{+} cells in the T1R1^{+} cells in the palate (T1R1-P) and floor of oral cavity (T1R1-F). Individual values for each chick were shown as white open circles. Values are the means ± SE. Absolute n number, number of taste buds, and number of cells were listed in the table (*right*). **D:** Ratios of T1R1^{-} or T1R1^{+} cells in the T1R3^{+} cells in the palate (T1R3-P) and floor of oral cavity (T1R3-F). Individual values for each chick were shown as white open circles. Values are the means ± SE. Absolute n number, number of taste buds, and number of cells were listed in the table (*right*).
