# Supplementary material for: The umami receptor T1R1–T1R3 heterodimer is rarely formed in chickens
Source: Sci Rep. 2021 Jun 10;11:12318. doi: 10.1038/s41598-021-91728-9 (PMC8192514; doi:10.1038/s41598-021-91728-9)
Supplement: Supplementary file 2 — Supplementary Information 2. [file 41598_2021_91728_MOESM2_ESM.pdf]

The umami receptor T1R1-T1R3 heterodimer is rarely formed in chickens

Yuta Yoshida[1,2], Fuminori Kawabata[1,3*], Shotaro Nishimura[1], Shoji Tabata[1]

[1]Laboratory of Functional Anatomy, Faculty of Agriculture, Kyushu University, Fukuoka, Japan

[2]Department of Food and Life Sciences, Ibaraki University, Ami, Japan

[3]Physiology of Domestic Animals, Faculty of Agriculture and Life Science, Hirosaki University, Hirosaki, Japan

***Corresponding author**: Dr. Fuminori Kawabata, Physiology of Domestic Animals, Faculty of Agriculture and Life Science, Hirosaki University, 3 Bunkyo-cho, Hirosaki, Aomori 036-8561, Japan. Tel.: +81-172-39-3805. Fax: +81-172-39-3805.
Email: kawabata@hirosaki-u.ac.jp

```
RJF    1  MIPWVLLCMSFGCAAALKPSCLSAQFRRPGDYIIGGLFPFGMDTINLTARSEPTLIVCERLFVDGLIWALGMKFAIDEINNSTSLLPGVE  90
WL     1  MIPWVLLCMSFGCAAALKPSCLSAQFRRPGDYIIGGLFPFGMDTINLTARSEPTLIVCERLFVDGLIWALGMKFAIDEINNSTSLLPGVE  90
RIR    1  MIPWVLLCMSFGCAAALKPSCLSAQFRRPGDYIIGGLFPFGMDTINLTARSEPTLIVCERLFVDGLIWALGMKFAIDEINNSTSLLPGVE  90

RJF   91  LGYDIYDTCFEPLAALQPSLLFVTQNGTTGIGIACNYTDYQPRVTAVIGPHKSDLCLLTAKLFSFFLIPQVSYGASSEKLSNKELYPSFY  180
WL    91  LGYDIYDTCFEPLAALQPSLLFVTQNGTTGIGIACNYTDYQPRVTAVIGPHKSDLCLLTAKLFSFFLIPQVSYGASSEKLSNKELYPSFY  180
RIR   91  LGYDIYDTCFEPLAALQPSLLFVTQNGTTGIGIACNYTDYQPRVTAVIGPHKSDLCLLTAKLFSFFLIPQVSYGASSEKLSNKELYPSFY  180

RJF  181  RTVPSDKNLVEAVVLLLDEFGWNWIATIGSDDEYGRGAQELFLSTIGNSSICIAYEGLIPSDLTDPRAEKQLEETIQYINKTNVNIIVLF  270
WL   181  RTVPSDKNLVEAVVLLLDEFGWNWIATIGSDDEYGRGAQELFLSTIGNSSICIAYEGLIPSDLTDPRAEKQLEETIQYINKTNVNIIVLF  270
RIR  181  RTVPSDKNLVEAVVLLLDEFGWNWIATIGSDDEYGRGAQELFLSTIGNSSICIAYEGLIPSDLTDPRAEKQLEETIQYINKTNVNIIVLF  270

RJF  271  AFRQPAQALLKQSIKMRLSKKVWIGTEAWLLSDIAASIPNIQNIGTVLGFIMKASTVPGFQKYVANLLSSVQQDEFCQKSRGFYRHVSSD  360
WL   271  AFRQPAQALLEQSIKMRLSKKVWIGTEAWLLSDIAASIPNIQNIGTVLGFIMKASTVPGFQKYVANLLSSVQQDEFCQKSREFYRHVSSD  360
RIR  271  AFQQPAQALLEQSIKMRLSKKVWIGTEAWLLSDIAASIPNIQNIGTVLGFIMKASTVPGFQKYVANLLSSVQQDEFCQKSREFYRHVSSD  360

RJF  361  TLGTQCQQCDHISLNDISSTLSHSQIQPVYIAVYSVAYALHRALGCTHQGCPRASIRSWQLLHFMNTVPFTVNGQSFRFDESHGTNSGYK  450
WL   361  TLGTQCQQCDHISLNDISSTLSHSQIQPVYIAVYSVAYALHRALGCTHQGCPRASIRSWQLLHFMNTVPFTVNGQSFRFDESHGTNSGYN  450
RIR  361  TLGTQCQQCDHISLNDISSTLSHSQIQPVYIAVYSVAYGLHRALGCTHQGCPRASIRSWQLLHFMNTVPFTVNGQSFRFDESHGTNSGYK  450

RJF  451  LIFWHWENGSLTHLPVGDYQESLYLNKSLIQFHTTDQKEPTSECFRECEPGQIRQIKGFHLCCYDCTDCPENTFCSSKDSSTCTPCLEHQ  540
WL   451  LIFWHWENGSLTHLPVGDYQESLYLNKSLIQFHTTDQKEPTSECFRECEPGQIRQIKGFHLCCYDCTDCPENTFCSSKDSSTCTPCLEHQ  540
RIR  451  LIFWHWENGSLTHLPVGDYQESLYLNKSLIQFHTTDQKEPTSECFRECEPGQIRQIKGFHLCCYDCTDCPENTFCSSKDSSTCTPCLEHQ  540

RJF  541  WSPARSTQCYDRSERYLRWNEPLTAGLLISMSIIISLICLTAVLFVKNLNTPLVQAAGGNLNLFALFALTLMCLSSCLFIGKPTNNLCMM  630
WL   541  WSPARSTQCYDRSERYLRWNEPLTAGLLISMSIIISLICLTAVLFVKNLNTPLVQAAGGNLNLFALFALTLMCLSSCLFIGKPTNNLCMM  630
RIR  541  WSPARSTQCYDRSERYLRWNEPLTAGLLISMSIIISLICLTAVLFVKNLNTPLVQAAGGNLNLFALFALTLMCLSSCLFIGKPTNNLCMM  630

RJF  631  QQIVCALCLNACFSTFFIKSLEIVLLTEFPRCARTALRWVTPSRSWLLVALCLLTECLFCFCYLHLGPDYVLPDYSSLPTEVLLMCSTAS  720
WL   631  QQIVCALCLNACFSTFFIKSLEIVLLTEFPRCARTALRWVTPSRSWLLVALCLLTECLFCFCYLHLGPDYVLPDYSSLPTEVLLMCSTAS  720
RIR  631  QQIVCALCLNACFSTFFIKSLEIVLLTEFPRCARTALRWVTPSRSWLLVALCLLTECLFCFCYLHLGPDYVLPDYSSLPTEVLLMCSTAS  720

RJF  721  WPAFALMHGYNGCLAFVCFLCTFMVQSSGKKYNMARGITFTILIYFIIWIFFITVFATLRTVLMSVIQISTILMVSLGIVGTYYIPKCYI  810
WL   721  WPAFALMHGYNGCLAFVCFLCTFMVQSSGKKYNMARGITFTILIYFIIWIFFITVFATLRTVLMSVIQISTILMVSLGIVGTYYIPKCYI  810
RIR  721  WPAFALMHGYNGCLAFVCFLCTFMVQSSGKKYNMARGITFTILIYFIIWIFFITVFATLRTVLMSVIQISTILMVSLGIVGTYYIPKCYI  810

RJF  811  LLLKPDLNREDYFQYSTKEEPEGDSQ  836
WL   811  LLLKPDLNREDYFQYSTKEEPEGD-   834
RIR  811  LLLKPDLNREDYFQYSTKEEPEGDPQ  836
```

Supplementary Figure S2

Supplementary Fig. S2. Amino acid sequences of chicken *T1R3* are aligned among 3 chicken strains, Red Jungle Fowl (RJF) obtained in the NCBI databases, White Leghorn (WL) obtained in the previous report [13], and Rhode Island Red (RIR), obtained in this study. Amino acid changes among strains are shown in black, and epitope sequence for the new antiserum generated in the present study is shown in red.
