# Supplementary material for: The umami receptor T1R1–T1R3 heterodimer is rarely formed in chickens
Source: Sci Rep. 2021 Jun 10;11:12318. doi: 10.1038/s41598-021-91728-9 (PMC8192514; doi:10.1038/s41598-021-91728-9)
Supplement: Supplementary file 3 — Supplementary Information 3. [file 41598_2021_91728_MOESM3_ESM.pdf]

The umami receptor T1R1-T1R3 heterodimer is rarely formed in chickens

Yuta Yoshida[1,2], Fuminori Kawabata[1,3*], Shotaro Nishimura[1], Shoji Tabata[1]

[1]Laboratory of Functional Anatomy, Faculty of Agriculture, Kyushu University, Fukuoka, Japan

[2]Department of Food and Life Sciences, Ibaraki University, Ami, Japan

[3]Physiology of Domestic Animals, Faculty of Agriculture and Life Science, Hirosaki University, Hirosaki, Japan

***Corresponding author**: Dr. Fuminori Kawabata, Physiology of Domestic Animals, Faculty of Agriculture and Life Science, Hirosaki University, 3 Bunkyo-cho, Hirosaki, Aomori 036-8561, Japan. Tel.: +81-172-39-3805. Fax: +81-172-39-3805.
Email: kawabata@hirosaki-u.ac.jp

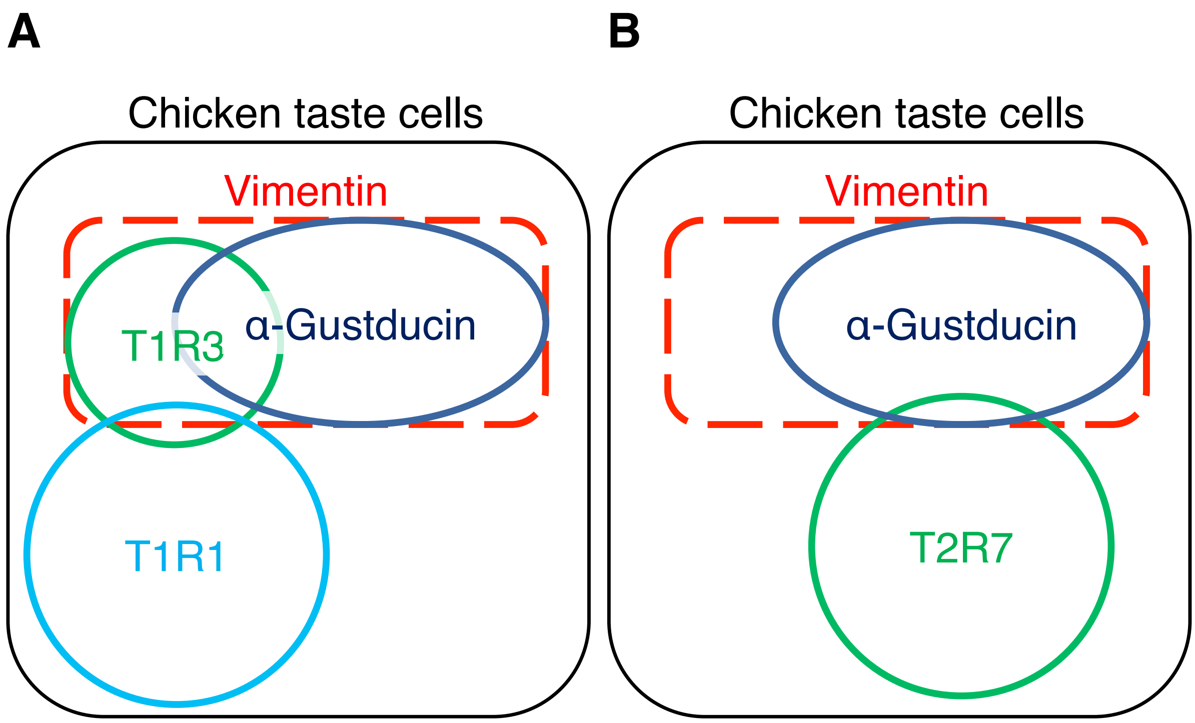

Supplementary Figure S3

Supplementary Fig. S3. A: Summary of the possible patterns of co-expression among vimentin, α-gustducin, T1R1, and T1R3 in the taste buds of chickens. T1R1 (blue circle) was mostly expressed in vimentin-negative taste cells, while T1R3 (green circle) and α-gustducin (navy blue circle) were largely expressed in vimentin-positive taste cells (red dashed area). **B:** Summary of the possible co-expression patterns among vimentin, α-gustducin, and T2R7 in the taste buds of chickens. T2R7 (green circle) was mostly expressed in vimentin (red dashed area)-negative and α-gustducin (navy blue circle)-negative taste cells.
